# Clinical validity of a population database definition of remission in patients with major depression

**DOI:** 10.1186/1471-2458-10-64

**Published:** 2010-02-11

**Authors:** Antoni Sicras-Mainar, Milagrosa Blanca-Tamayo, Laura Gutiérrez-Nicuesa, Jordi Salvatella-Pasant, Ruth Navarro-Artieda

**Affiliations:** 1 Directorate of Planning, Badalona Serveis Assistencials SA, Badalona, Barcelona. Spain, Gaietà Soler, 6-8 entlo. 08911 Badalona. Barcelona. Spain; 2 Psychiatric Services, Badalona Serveis Assistencials SA, Badalona, Barcelona. Spain; 3 Medical Department, Lundbeck España SA, Barcelona. Spain; 4 Medical Documentation, Germans Trias i Pujol Hospital, Badalona, Barcelona. Spain

## Abstract

**Background:**

Major depression (MD) is one of the most frequent diagnoses in Primary Care. It is a disabling illness that increases the use of health resources. Aim: To describe the concordance between remission according to clinical assessment and remission obtained from the computerized prescription databases of patients with MD in a Spanish population.

**Methods:**

Design: multicenter cross-sectional. The population under study was comprised of people from six primary care facilities, who had a MD episode between January 2003 and March 2007. A specialist in psychiatry assessed a random sample of patient histories and determined whether a certain patient was in remission according to clinical criteria (ICPC-2). Regarding the databases, patients were considered in remission when they did not need further prescriptions of AD for at least 6 months after completing treatment for a new episode. Validity indicators (sensitivity [S], specificity [Sp]) and clinical utility (positive and negative probability ratio [PPR] and [NPR]) were calculated. The concordance index was established using Cohen's kappa coefficient. Significance level was p < 0.05.

**Results:**

133 patient histories were reviewed. The kappa coefficient was 82.8% (confidence intervals [CI] were 95%: 73.1 - 92.6), PPR 9.8% and NPR 0.1%. Allocation discrepancies between both criteria were found in 11 patients. S was 92.5% (CI was 95%: 88.0 - 96.9%) and Sp was 90.6% (CI was 95%: 85.6 - 95.6%), p < 0.001. Reliability analysis: Cronbach's alpha: 90.6% (CI was 95%: 85.6 - 95.6%).

**Conclusions:**

Results show an acceptable level of concordance between remission obtained from the computerized databases and clinical criteria. The major discrepancies were found in diagnostic accuracy.

## Background

Major depression (MD) is one of the most frequent diagnoses in Primary Care (PC) and in the general population [1]. It is a disabling illness that alters patients' quality of life and causes an increase in the use of health resources [2-6]. It is important to define the therapeutic objectives for MD [7], and more experts are claiming that reaching remission should be the main objective since residual symptoms prolong psychosocial dysfunction and can cause higher rates of recurrence [8-10]. In this respect, antidepressants (AD) constitute the pillar of pharmacological treatment in order to achieve the sustained remission of symptoms [11,12].

On the other hand, some prospective naturalistic studies are carried out in order to explore health problems using computerized databases; because key data in these databases are prescriptions or pharmacy claims, and because there is no specific clinical data recorded in these databases it is usually not possible to obtain quality data to measure remission, thus necessitating "approximate definitions". No consensus currently exists regarding a definition of an approximation for remission. However, definitions exist for depression episode: usually, a new episode for depression is defined as having a new antidepressant prescription with no antidepressant prescription in the previous 6 months [12]. From this, it can be derived that if a patient stops treatment and has no new prescription during the following 6 months, he/she has completed a depression episode. If he/she starts an antidepressant after that, it will be a new episode. This can be used in a model for remission.

Nevertheless, this "approximate definition" can cause several problems. On one hand, there may be other motives for discontinuing the prescription other than the patient being in remission (failure to observe treatment, adverse side effects, etc.). On the other hand, persistence of treatment with AD should not always be interpreted as absence of remission, as it is indicated in all patients for 6-9 months after achieving remission, and for a longer period in selected patients at risk of relapse [13].

Thus, it is pertinent to prove the validity of this construct, so as not to fall into classification bias [14], especially when there is no evidence at present in the revised literature. This study is an attempt to complement a previously started line of investigation on remission in patients with MD (results pending publication). The purpose of this study is to describe the validity (reliability, concordance) and the clinical utility of the remission measurement obtained from the direct review of patients' histories (reference criteria) and the one taken from the database registry (by approximation) in MD patients in a Spanish population.

## Methods

### Design and population under study

The design was cross-sectional and multi-centric. The population under study was comprised of people from six primary care facilities under the management of Badalona Serveis Assistencials SA, which provide health care for some 107,208 people, out of whom 15.9% are over 64. The diagnosis of MD was made according to International Classification of Primary Care (ICPC-2; code P76) [15]. The following scales were used for screening/diagnostics: Goldberg (co-existence of psychiatric morbidity), Hamilton Depression (severity of depression symptoms) and Yesavage Geriatric Depression [11]. Patients were selected according to the following criteria: a) over 17 years old, b) who have initiated a first MD episode with a treatment prescribed between January 2003 and March 2007 (determined for diagnosis by clinical interview of treating clinicians according to the ICPC-2), c) and demonstrate a period of at least six months without depression prior to the MD episode, d) prescription should follow the minimum required treatment criteria [11] (> 60 days of AD treatment after the first prescription) and e) a follow-up of at least 18 months (12-month study period and 6-month follow-up to assign study sub-group). Two sub-groups were considered: patients in remission and patients not in remission.

### Definition of remission by approximation and according to reference criteria

Patients were considered to be in remission when they no longer required new prescriptions of AD for at least six months after completing treatment of the first episode; patients who could only interrupt treatment for a period less than six months were not considered in sustained remission [12]. As reference criteria, remission was determined based on the assessment of the database clinical course text by a psychiatry specialist. The sample was selected through simple random sampling stratified for age and gender. Sample size was calculated assuming an expected remission prevalence of 54%, a random error of 5% (bilateral) and an accuracy of 8.5%.

### Validity and utility measure indicators

Validity indicators were Sensitivity [S], specificity [Sp], positive and negative predictive value [PPV] and [NPV]) and clinical utility (positive and negative probability ratio [PPR] and [NPR]. S is the probability of obtaining a positive result when the subject meets the approximation definition. Sp indicates the probability of obtaining a negative result when the subject does not meet the approximation definition. PPV is the probability of meeting the reference criteria when the approximate remission variable is positive. NPV is the probability of not meeting the reference criteria when remission by approximation is negative. The probability ratios of positive or negative likelihood-ratios indicate up to what point a certain result in a diagnostic test will increase or diminish pre-test probability [16].

### Confidentiality of the information and statistical analysis

Data confidentiality was respected at all times, according to the Personal Data Protection Act (*Ley de protección de Datos de Carácter Personal *[LOPD]); this study was approved by the Ethics Committee of Gol i Gurina Foundation. Concordance between both remission measurements was evaluated, itemized by systematic error and random error. Concordance index was calculated using Cohen's kappa coefficient, correlation coefficient was calculated for random error and McNemar test for systematic error [17]. Reliability analysis was established using Cronbach's alpha. Confidence intervals [CI] of 95% were calculated. SPSSWIN version 12 was used, establishing a statistic significance of p < 0.05.

## Results

4,572 subjects, according to the database criteria, complied with the selection (new episode of depression), of whom 54.6% (CI of 95%: 53.2 - 56.0%) were considered to be in remission. 133 patient histories were reviewed according to clinical criteria. Concordance between remission by approximation and remission according to reference criteria is detailed in table 1. The weighted kappa concordance index obtained was 82.8% (CI of 95%: 73.1 - 92.6), PPR 9.8% and NPR 0.1%. Eleven patients showed discrepancies between remission by approximation and remission according to reference criteria. In five cases (false positives), review of the clinical course revealed they did not meet the clinical criteria for remission, the cause of this discrepancy was either incorrect initial diagnostic classification or on later occurrence of stress factors (dysthymia: 2; recurrent depression: 1; adaptive disorder: 1; and vital occurrence: 1). Six patients (false negatives) were in remission by clinical course assessment but not approximation criteria. These patients were wrongly classified (dysthymia: 4; adaptive disorder: 1; recurrent depression: 1) and required treatment to prevent recurrence for a longer period of time.

**Table 1:** Validity of remission by approximation in major depression

Validation Method	Reference Criteria	
Remission by Approximation	Clinical Histories	
N = 133	Positive	Negative
Positive	74	5
Negative	6	48

Statistics	Value (%)	95% CI

*Validity of the measurement*		
Sensitivity	92.5	88.0 - 96.9
Specificity	90.6	85.6 - 95.6
Positive predictive value	93.7	89.6 - 97.8
Negative predictive value	88.9	83.6 - 94.2
False positives	7.5	3.0 - 11.9
False negatives	9.4	4.4 - 14.4

*Reliability*		
Cronbach's alpha	90.6	85.6 - 95.6
Area under the curve	91.2	86.5 - 96.1

*Concordance*		
McNemar test	58.1	49.7 - 66.5
Pearson correlation	82.8	73.1 - 92.5
Weighted kappa (Cohen)	82.8	73.1 - 92.6

*Clinical utility*		
Positive Probability Ratio	9.8	---
Negative Probability Ratio	0.1	---

Reference criteria: Review of patients' histories. Significance: p < 0.001 in all cases. CI: confidence intervals

## Discussion

Our study deals with the clinical validity of the measurement of remission in patients with major depression, as obtained from population databases. It is worth pointing out that the organization of care, through territorial-based assignment of teams and the increasing computerization of its centres provides a suitable environment to carry out these types of studies in a clinical practice environment [18,19]. Thus, the quality of the measurements is a basic aspect in achieving an efficient health system, and we must be sure in any kind of biomedical research that measurement error is reasonably small. In regulated environments, such as clinical testing in the development of pharmaceuticals, data quality and especially measurement procedures require attention for reasons related to both ethics and efficiency [17].

The results of our study reveal an acceptable degree of concordance between the remission by approximation and the variable obtained from patient history reviews (reference criteria). The main cause of discrepancy between them is diagnostic accuracy by the PC team. In this respect, several authors have affirmed the existing variability between the PC diagnosis and the one by mental health specialists [20-22]. There seems to be certain difficulty in identifying affective and adaptive disorders, which has an impact on remission measurement. There are several possible motives affecting MD diagnosis. On one hand, it may be affected by shortcomings in the training of the professionals, health care pressure in their consults and the brief amount of time they can devote to each patient.

Furthermore, it can also affect the classification system used (ICPC-2), which can condition the diagnosis of these patients. In this regard, it must be taken into consideration that these differences between both disorders make differential diagnoses difficult, as PC doctors usually tend to view disorders from a dimensional rather than a categorical perspective, probably because it best fits the needs of their consultation. Nevertheless, there are studies that reveal a certain tendency of over-diagnosis of depression disorders, while others point out an under-diagnosis of MD and anxiety in PC [23,24]. The interpretation of the measurement of remission in our study may be in agreement with the interpretation of probability coefficients (PPR and NPR) as the measurement of clinical utility. Thus, the measurement of remission by approximation can generate moderate changes from pre-test to post-test probability (reference criteria).

Possible limitations in the study demand caution in generalization of results. The study presented is framed within the studies known as technique validation in clinical effectiveness conditions; therefore, the bias assumed in research is typical of a study with a cross-sectional model, oriented towards validation of diagnostic criteria. Moreover, sample size can have a high random component for precision in measurement, and the study was carried out based on a specialist's diagnostic criteria; therefore, the results obtained may be influenced by the professional's characteristics.

## Conclusions

Results show acceptable concordance in the remission obtained from the computerized databases. The largest discrepancies are in diagnostic accuracy.

## Abbreviations

MD: Major Depression; PC: Primary Care; AD: Antidepressants; ICPC-2: International Classification of Primary Care; S: Sensitivity; Sp: Specificity; PPV: Predictive Positive Value; NPV: Negative Predictive Value; PPR: Positive Probability Ratio; NPR: Negative Probability Ratio.

## Competing interests

Research was funded by Lundbeck Laboratories SA, with no influence in the final results.

## Authors' contributions

MB and LG organised the bibliographical research. AS and MB obtained data from review of the patients' histories. AS and RN conducted the analysis and interpretation of results. All the authors contributed ideas, interpreted findings, revised and approved the final version of the paper. AS is the main responsible for this study.

## Pre-publication history

The pre-publication history for this paper can be accessed here:

http://www.biomedcentral.com/1471-2458/10/64/prepub
